# “I Wouldn’t Be Friends with Someone If They Were Liking Too Much Rubbish”: A Qualitative Study of Alcohol Brands, Youth Identity and Social Media

**DOI:** 10.3390/ijerph15020349

**Published:** 2018-02-16

**Authors:** Richard I. Purves, Martine Stead, Douglas Eadie

**Affiliations:** Institute for Social Marketing and UK Centre for Tobacco & Alcohol Studies, Faculty of Health Sciences & Sport, University of Stirling, Scotland FK94LA, UK; martine.stead@stir.ac.uk (M.S.); douglas.eadie@stir.ac.uk (D.E.)

**Keywords:** alcohol, branding, identity, young people, social media, marketing

## Abstract

The consumption of alcohol by young people remains a major public health concern at both the national and international level. Levels of drinking among 15-yearolds in the United Kingdom (UK) remain significantly higher than the European average. This study explored how alcohol brands are used by young people to develop their desired identities and how these acts of consumption extend to young people’s profiles on social media. It also deepens understanding of how alcohol brands are connected to young peoples’ concerns about image and peer group dynamics. This involved qualitative focus groups with young people aged 14–17 in Central Scotland. Certain alcohol brands were approved and viewed as socially acceptable by young people, while others were rejected. Children as young as 14 were selecting products to portray a drinking identity that was appropriately aligned to their gender and sexuality. Participants displayed a desire to associate themselves with the mature drinking culture personified by some brands, whilst simultaneously distancing themselves from immature drinking practices associated with others. Publicly associating with alcohol brands on social media carried with it potential risks to peer group acceptance. Understanding how young people perceive alcohol brands, the importance of social media in communicating that identity to their peers and the role that alcohol brands play in adolescent identity formation is an important first step to reforming alcohol marketing regulations.

## 1. Introduction

The consumption of alcohol by young people remains a major public health concern at both the national and international level. It has been linked with poor educational performance, risky sexual behaviour and teenage pregnancy [1,2], crime and disorder [3,4] and a range of physical and psychological harms [5,6]. In addition, research has shown that the earlier a young person begins to drink alcohol, the greater the likelihood that they will develop alcohol dependency later in life [1,7]. Although recent surveys have found that young people in the UK are drinking less often during the week, the proportion of 16–24-year-olds drinking at least twice the recommended number of units in a session at least once a week remains above the total average for both sexes [8]. Statistics from England show that, although the average amount of alcohol consumed by 11–15-year-olds appears to be declining, around 8% of the total population of 11–15-year-olds consumed alcohol in the last week. Around half of these young people drank six or more units, with 22% drinking 15 units or more [9]. Prevalence of regular drinking has been shown to increase with age, from less than 0.5% of 11-yearolds reporting drinking at least once a week to 10% of 15-year-olds [10]. Current guidelines produced by the Chief Medical Officers specify that males and females should drink no more than 14 units of alcohol per week and to spread this evenly over three days or more [11]. The previous guidelines were 21 units for men and 14 units for women per week. This level of drinking remains a cause for concern, particularly since most adolescents consume the maximum (or greater) number of recommended drinks per week in one or two days resulting in what has been described as a ‘two-tier’ drinking culture [12]. Levels of drinking among 15-year-olds in the United Kingdom also remain significantly higher than the European average [13]. This pervasive social norm of heavy drinking has been referred to as ‘the culture of intoxication’ and research indicates the association between (heavy) drinking, pleasure and fun for young people [14,15]. Research has also found that young people view the act of consuming alcohol as an outward projection of maturity [4].

Adolescence can be a period of transition in which young people seek to establish their personal identity [16]. Within the context of peer group culture, young people must learn how to present themselves as the same or as different from others and to manage the tensions between conformity and individuality [17]. Young people’s identities are entrenched in complex networks of relations where the power to include or exclude others is central to how they relate to each other. Therefore, the act of consumption takes on new significance in adolescence. Instead of being entirely dependent on their parents making choices for them, young people now have independent entry into social and cultural life through the consumption of fashion, leisure and material goods. Adolescents will use consumer goods as an outward expression of self-identity. In addition, the products a person consumes lead others to make judgements about their personality [18]. Consumption plays a key role in shaping young people’s identities and how these are expressed and navigated in their social lives [19].

Youth culture attaches great value to brand labels and symbols and young people use brands to help present their desired identity to others [20]. However, it is equally important for young people that they be seen to be consuming what their peer group would consider ‘the right brands’ [21]. Branding plays an important role in young people’s consumer behaviour since they encapsulate the perceived value of attributes of products. Marketers build intangible emotional benefits into their brands in a bid to evoke certain feelings when consumers use their product. In essence, the brand becomes an extension of the consumers’ self-identity through a process known as ‘symbolic self-completion’ whereby the meanings and values of the brand transfer to the individual who is seen to be consuming it [22,23]. Brands seek to become intimately connected to consumers’ social practices in order to produce a particular relationship to the brand and to manage this relationship in ways that will add value to the brand [24,25]. Previous research into brands’ use of social media argues that, rather than simply impose particular meanings, brands have moved toward managing consumer activity around cultural events and social spaces provided by the brand [26,27]. From this perspective, branding is a social process that incorporates the participation of consumers [24,28]. Involving consumers in marketing in order to produce a dimension of trust or authenticity to the messages is a frequently used strategy on social media. Carah et al. argue that brands are less concerned with controlling, and being responsible for meanings online. Rather they concentrate on enhancing and managing the way the brand is spread throughout social networks, blurring the distinction between commercially generated and user-generated content [28].

Consumer research also suggests that young people will consciously avoid certain brands when the brand image is symbolically incongruent with the individual’s identity [29]. Therefore, the consumption of brands helps adolescents create and present a desired identity as well as helping them fit in with their desired peer group [30]. Previous research has examined how the process of buying branded goods or following particular fashion trends impacts on image and social status [31,32]. Existing literature has also explored the values young people attach to certain food and tobacco brands [30,33,34,35] and the impact of alcohol marketing and branding on young peoples’ alcohol consumption [36,37]. Recent research from New Zealand has focused on the central role of alcohol in young people’s social media practices and interactions [38,39].

This study set out to build on this research, and to explore how these acts of consumption extend to young people’s online behaviour on social networking sites. It also sought to deepen understanding of how alcohol marketing is connected to young peoples’ concerns about image and peer group dynamics.

The internet is an area where exposure of young people to alcohol marketing is potentially high [40]. Social media is an important part of the alcohol industry’s multi-platform marketing strategies [41,42] and research has identified a number of creative strategies used by the alcohol industry to advertise their products through social media [39,43,44]. Recent studies have found associations between engagement with, and awareness of, alcohol marketing on social media and intentions to drink [45] more frequent alcohol consumption [46,47] and heavy episodic drinking [48]. The large volume of alcohol-related content identified on social media such as Facebook and Twitter have been highlighted as an area of growing concern regarding the inadequacies of current regulations to protect young people from exposure to alcohol marketing [41,44,49]. Although age restrictions on certain social media sites prevent some young people from interacting with alcohol marketing through the use of ‘age affirmation’ gates where the user confirms they are a certain age, these are often easily circumvented and have been criticised for being too lax [47,50]. In addition, young people can be exposed to alcohol marketing based on their interactions with the social media pages of certain music or sporting events or as a result of their friend’s interactions [43,51,52]. Although many adults use social media, research has found that online social networking is an important part of youth culture which facilitates participation in a peer network and the development of social identity [53,54,55]. Social media offers new avenues for young people to create ‘consumer identities’ through engagement with brands [56]. Social media also helps in the creation and maintenance of certain social norms and behaviours [57]. This is particularly important when it comes to alcohol use because research has identified alcohol expectancies and perceptions of peer consumption habits as predictors of alcohol use [49]. Alcohol content on social media is generally positively depicted which influences perceived drinking norms [58]. Recent research has focused on the central role of alcohol in young people’s social media practices [44,52] and the impact this has on alcohol-related norms [51,59,60,61]. The research reported here sought to explore these themes further by focusing on how alcohol brands are used by young people to develop their desired identities and the role that social media plays in this development. We conducted qualitative research to explore the relationship between alcohol brands and young people’s identities and how this relates to their profiles on social media.

## 2. Materials and Methods

### 2.1. Design

We conducted qualitative focus groups with young people aged 14–17. Focus groups are effective ways of exploring how established groups interact in relation to the phenomenon under investigation, thus they are particularly valuable for looking at social phenomena such as how brands can be used to communicate identity and contribute to peer acceptance. Ethical approval for conducting this research was granted by the University of Stirling research ethics board (Application No. 39). Eight focus groups (6 participants per group, *n* = 48) were conducted involving single--sex friendship groups of girls and boys recruited in two categories defined in a previous study on adolescent drinking: 14–15-year-old ‘starter drinkers’ for whom drinking is becoming more common and frequent; and 16–17-year-old ‘established drinkers’ whose social lives quite regularly involve drinking and in different environments such as pubs and nightclubs [62]. Many authors claim that single-sex groups work best for children and teenagers as they allow young people to feel more comfortable in reasonably homogenous groups [63]. Groups were also divided according to socio-economic group, based on the occupation of the main income earner in their household [64] (See Table 1). Groups were recruited face-to-face by independent consultants using a detailed recruitment questionnaire which asked their age, gender, socio-economic group and their alcohol consumption within the last year.

All participants had consumed alcohol at least once a year or less (9 consumed alcohol at least once a week, 11 consumed alcohol once a month, 8 consumed alcohol at least twice a year and 12 consumed alcohol once a year or less). Ethnicity of participants was not considered. In order to recruit friendship groups, consultants initially contacted one index individual who met the criteria specified in the recruitment questionnaire. They were then requested to invite friends conditional on them also meeting the criteria specified (same gender, age group, had consumed alcohol within the last year). This meant that some groups consisted of more than one group of friends. Participants were also asked which social media sites they visited (Facebook, Twitter, YouTube, Tumblr, Pinterest, Other) and how often they visited them (several times a day, once a day, several times a week, once a week, once a month, less than once a month). Facebook was the most popular social media site amongst the young people with 36 participants (75%) reporting to visit it several times a day, 10 participants reporting to visit it once a day, 1 participant visited it several times a week and 1 visited it less than once a month (Table 2).

An information letter that outlined the nature of the study was provided to parents and participants. The focus groups took place in January and February 2014 and were arranged at times so that participants could attend after school. Groups were held in informal community settings in the cities of Edinburgh and Glasgow and participants were recruited from the immediate locality. Written consent was obtained from both participants and their parents and groups were audio-recorded with participants’ consent. Twenty-two examples of alcohol packaging were available for participants to handle during the focus groups and further examples of alcohol marketing were shown using a tablet device. This included the brands’ websites as well as their official Facebook, Twitter and YouTube pages (where these existed). These examples were selected based on industry data on the top 100 alcohol brands in the UK [65]. Content from branded social media pages and websites was used to facilitate discussion and encourage participants to talk about socialising with reference to alcohol and marketing. Two male researchers (RP and DE) individually moderated the groups which lasted between 60 and 90 min. The research team had no prior relationship to the participants.

### 2.2. Analysis

As we were examining a relatively new phenomena it was important to use an inductive, open-ended approach to gain the depth necessary to developing new meaning and understanding, otherwise the research could risk simply repeating or confirming what was already known. Analysis of transcripts was led by one researcher (RP), and a set of themes based on the core questions and topic areas was agreed among the research team [66]. The reliability of these themes was then reassessed by a process of familiarization with the transcript texts and cross-examination. Discussions between researchers enabled identification of emerging themes and resolution of interpretive difference. These analyses allowed the investigation team to identify patterns across the data as a whole.

## 3. Results

We focus here on three key themes which emerged from the data. Firstly, that certain alcohol brands are approved and viewed as socially acceptable by the young people due to their associations with particular gender, age and sexuality while others are rejected. Secondly, that their choice of alcohol brand can be used as an indicator of their identity which can be used to fit in with their desired peer group. Thirdly, that the values and meanings attached to alcohol brand choices extend to social media which acts as a public platform where open endorsement of brands by ‘liking’ or sharing their marketing can carry with it significant social risks.

These themes are now discussed more fully with regard to three alcohol brands in particular which were discussed at length in the focus groups: Budweiser, Malibu and WKD (See Table 3 for more details). These three brands were among the 22 examples of product packaging used as prompts for the focus groups. The paper here focuses on these three brands as they were the most commonly cited by participants in all 8 groups and all groups expressed some level of familiarity with these brands. They also represent three different beverage categories (Table 3) and were used by the participants to demonstrate particular drinking identities. It is therefore not clear to what extent their talk about these brands and about their social media profiles reflected their actual engagement with alcohol brands online, their drinking practices and/or their social media practices. However, it does reveal their perceptions of specific alcohol brands and how this relates to the construction of their social media profiles as a key element of youth identity formation. Pseudonyms have been used to delineate which respondent is speaking in verbatim quotes.

### 3.1. Alcohol Brands and Portraying Identity

In our focus groups, the young people spoke at length about certain alcohol brands being approved by their peers and viewed as socially acceptable, while others could be potentially damaging to one’s image. As detailed above, participants were shown examples of alcohol packaging and asked to describe their perceptions of the brand, who they thought the product was aimed at and the values they associated with people who consumed the brand.

For the young people in the focus groups, gender emerged as an important dimension for establishing the acceptability of certain brands within their peer group. Alcohol brands could be used to signal something about yourself to others. For example, it was widely believed that Budweiser had strong male associations and was targeted at the male football fan. The overriding view from the male and female participants was that Budweiser and other lagers were brands which were widely known amongst their peer group due to their associations with football (e.g., Budweiser was associated with the Fédération Internationale de Football Association (FIFA) World Cup and Football Association (FA) Cup football tournaments). This brand appealed to the young males because they felt they would not be stigmatized or excluded for consuming them. By contrast, Malibu was viewed as a very feminine product which was exclusively consumed by females. This is in line with previous research which has shown how alcohol is used to enact forms of masculinities and femininities [67,68,69].

Further discussion revealed that the acceptability of alcohol brands depended on associations with both gender and age and the implications this had for the way people consumed these brands. For example, Malibu was viewed as a drink for females either in their late teens or early-twenties who would consume it whilst out socializing with their friends:

Becky: “Just because it looks like a young like, not a young, but a person who is in their thirties or twenties would drink it before going out.”

Moderator: “Before going out, ok, so what do they like to do?”

Alison: “It’s like, it looks like kind of girly nights in and stuff, like if they were going out like what you (Becky) said.”

Moderator: “So a girly drink?”

Alison: “Yeah.” (Female, 14–15, ABC1)

The young females aspired to this type of social drinking as it was viewed as an acceptable lifestyle for young women. This discussion also highlighted the importance of socio-economic status to the acceptability of certain brands, particularly for those young females classified as ABC1. Malibu was perceived as a drink for young women from a higher income bracket who were able to afford to regularly go out to bars and night clubs. Therefore, if you were seen to be consuming this brand it would lead others to make certain assumptions about your personality, lifestyle and socio-economic status. The young females referred to “cocktail nights” (Jane, Female, 14–15, ABC1) and “going out with their friends” (Louise, Female, 16–17, C2DE) as a desirable lifestyle. Malibu was associated with this type of mature, feminine drinking identity: “That’s what I think of (when I see that product), going out and having a great time with your friends” (Jane, Female, 14–15, ABC1). Drinking spirits and cocktails was viewed as a more feminine thing to do than drinking beer and Malibu was viewed as an acceptable brand for a female to be seen with and was not deemed an acceptable brand of choice for males:

“Men will drink beer, our age. This is more girly sort of stuff.”

(Karen, Female, 16–17, ABC1)

In much the same way, Budweiser was particularly appealing for the young males in the sample who wished to present an image of a more mature, self-assured drinker who enjoyed drinking for pleasure as opposed to a product such as vodka which was associated with binge drinking and immaturity. Both males and females believed that “older guys drink it” (Linda, Female, 14*–*15, C2DE), and this identity was desirable for the young males:

Rob: “You are seen drinking vodka it’s sort of like it’s like you are trying to set the whole binge drinking vibe aye, but if you are drinking Bud.”

Steve: “Going out to get drunk.”

Rob: “Budweiser it’s not like you are.”

Mark: “Just relaxing eh.”

Steve: “Yeah it’s not like you are, yeah it’s not like you are trying to get drunk.”

Mark: “More laid back.”

Steve: “Yeah it’s a different sort of drink.” (Males, 14–15, ABC1).

In this sense the male participants aspired to a more relaxed style of alcohol consumption as it aligned with their desire to project an outward appearance of maturity and control, and not having to try too hard to fit in. This discussion also incorporated elements of social allegiance where a relaxed, non-binge style of drinking was associated with a higher socio-economic status.

### 3.2. Using Alcohol Brands to Fit in

The examples above demonstrate how perceptions of age and gender interact and influence how young people accept brands as a means of constructing a social identity. However, as the following case demonstrates, the rejection of brands is equally important to identity formation and can play an important role in conformity. Male participants stated that they would avoid products such as WKD as these were seen to be aimed at the opposite sex or regarded as ‘girly’ or ‘feminine’, and drinkers ran the risk of being insulted if they were seen consuming it:

David: “I don’t like WKD.”

Scott: “It’s the same strength as beer but tastes nicer”

Moderator: “So who would drink WKD then?”

David: “Girls.” (Male, 14–15, C2DE)

Such behaviour was viewed as socially unacceptable and could be detrimental to the young male’s image. However, WKD was also rejected by both males and females in the focus groups for being associated with those much younger than themselves. This product was thought to be designed to specifically appeal to younger drinkers and this was reflected in a belief that the product was designed to look and taste like a soft drink, “like a Fanta or a Coca Cola” (Derek, Male, 14–15, ABC1) and was derided for its low alcohol content. There was also an indication that WKD was also rejected due to its connotations with lower social-economic status. The drink was believed to be consumed by “wee neds” (Keith, Male, 16–17, ABC1), a term used to describe males or females from disadvantaged backgrounds. The rejection of WKD also served to demonstrate heterosexuality amongst the young males who described the product as “poof juice” (Peter, Male, 16–17, C2DE). Females also regarded consumption of WKD as something males would be criticized for, again highlighting the aspect of sexuality in their description of the product:

Ann: “It’s poof juice, that’s what it’s called, poof juice.”

Louise: “Because it’s the weakest kind of drink.” (Females, 16–17, C2DE).

Low alcohol drinks such as WKD were generally regarded negatively and were seen as ‘starter drinks’ for the very young who were striving for credibility, but were out of touch. Participants discussed how WKD might be purchased by parents so that their children could have a drink at special occasions:

Michael: “If you’ve got a young girl I’d say. If you are a parent then you’d give your daughter WKD.”

Scott: “Say its New Year and they are not old enough to.”

Moderator: “So it’s the sort of thing parents might buy their kids.”

Michael: “Aye, to get them into the game.” (Males, 14–15, C2DE).

Because these drinks were not popular amongst their peer group, participants generally distanced themselves from these products. Consequently, being associated with this brand carried significant social risks:

“If you go out and drink it (WKD) on the streets you will either get leathered for being a gimp, or you will just get the piss taken straight out of you.”

(Rachel, Female, 16*–*17, C2DE)

### 3.3. The Risks of Associating with the Wrong Brands on Social Media

Discussions revealed that the values and meanings attached to alcohol brand choices extended to participants’ online profiles. It was evident that for many of the young people the use of social media was a common feature of everyday life. Participants discussed alcohol marketing on several social media outlets (Facebook, Twitter, Instagram, Pinterest, Tumblr and YouTube). However, the findings here mainly focus on alcohol marketing on Facebook which, in addition to being the most discussed outlet, also facilitates an individual’s ‘likes’ and ‘shares’ to be known to their online contacts. Who and what a person associated themselves with on social media could influence their ability to make new friends and contacts therefore the brands ‘liked’ by respondents were often carefully selected. All were aware that once a user made a decision to ‘like’ a commercial page, this preference was available for all of their friends to see, so it was considered important that they associated themselves with what were considered accepted brands. Similarly, social media was also important to screening potential new friends and contacts, with the brands and organisations a person chose to ‘like’ or ‘follow’ on social media providing a way of making a judgement as to whether or not that person shared similar values:

Kate: “I wouldn’t be friends with someone if they were liking too much rubbish.”

Ann: “All my friends will be able to see what I like.”

Kate: “Yeah, so that’s even more like annoying because they can comment on that if they want to as well.” (Females, 16–17, C2DE).

While discussing motivations for interacting with alcohol marketing on social media, the general view was that it would be people who wanted to demonstrate that they consumed the product to their social network. In this sense, interacting with alcohol brands online carried with it certain social risks, most specifically that it could be viewed negatively by friends, particularly if it was an unpopular brand. For example, liking the Facebook page for WKD was widely regarded as an embarrassing thing to do, not just because the individual would be declaring that they drank WKD, but also because they would be indicating that they thought it was acceptable to do so. One female participant admitted to having liked the WKD Facebook page in the past, however, this admission carried with it a sense of shame in having liked something which was viewed negatively by her peers:

“Do you know what’s bad, I’ve liked that page … I think it I just liked it because of the colour of the drink.”

(Paula, Female, 16–17, C2DE)

She also recalled earlier conversations regarding the brand’s reputation as a drink for younger people as a way of justifying her association with WKD:

“It was the bottles and the colours and that was the first thing I ever got pissed on”

(Paula, Female, 16–17, C2DE)

Although the other females in this group accepted this as a justification for interacting with this brand, other groups indicated that showing allegiance to alcohol brands which were deemed to be socially unacceptable carried with it certain risks. For example, one group discussed the implications of posting a picture of themselves with a bottle of WKD on social media:

Scott: “WKD for girls is alright.”

Daniel: “If I see a guy on Facebook posting a picture with one of them (WKD), I’d delete him.”

David: “You wouldn’t want to be caught drinking that if you were a guy.”

Daniel: “Pelters thrown at you.”

Moderator: “So that would be social suicide?”

David: “That’s deleting your Facebook account sort of stuff.”

Daniel: “You wouldn’t want to put a picture up of a bottle of WKD.” (Male, 14–15, C2DE).

Although expressed in a light-hearted manner, accounts such as this did serve to demonstrate the importance of being associated with the right alcohol brand and its importance to social status. It also demonstrates the risks inherent in being associated with what are considered to be socially unacceptable brands due to their negative associations. Pictures of Budweiser were viewed more favourably by the male participants as this was seen as normal behaviour and more socially acceptable.

Scott: “You see pictures with folk with a bottle or a can of Bud on Facebook all the time.”

Michael: “Aye.”

Daniel: “That’s normal.” (Males, 14–15, C2DE).

Importantly, the negative attitudes towards alcohol marketing on social media softened somewhat when participants were invited to consider content from other brands which were viewed as more socially acceptable by their peer group. For example, Malibu’s social media communications were viewed far more favourably, particularly by the female participants. As this brand was more socially acceptable, the prospect of interacting with the brand was more appealing. The female participants found Malibu’s online cocktail recipes and consumption suggestions particularly appealing, again associating the brand with parties and socializing:

Margaret: “I think it’s quite good—what you can do with it.”

Joanne: “Maybe if people have parties to see what they can do with drink.” (Females, 16–17, ABC1).

By creating particular brand associations and displaying distinct personalities through their marketing activities, this influenced the social acceptability of the brand among the young people. Football featured heavily on Budweiser’s social media pages which were viewed very favourably by the young males who admitted they would be more inclined to interact with beer brands online without fear of ridicule:

John: “If you are into football then that page is pretty attractive … you’d be more likely to engage with it.”

Peter: “Yeah, that would be more acceptable than posting on WKD.” (Males, 16–17, C2DE)

One young male who had earlier admitted to liking and sharing football posts on his Facebook profile stated that he would happily like or share some of Budweiser’s football-related marketing content because it aligned with his desired identity:

Daniel: “Budweiser sponsor the FA Cup, I’ve just downloaded the app so I’d probably like their page as well to keep up to date with things.”

Moderator: “Would this be the sort of thing you would share with your friends?”

Daniel: “Yeah, probably. They’ll be able to see it if I like it but sometimes I’ll share things if they’re football-related” (Male, 14–15, C2DE).

## 4. Discussion

For adolescents, consumption choices can be used to create and reinforce the image they choose to project and to identify themselves with particular social or peer groups. Our study explored, through focus groups, the intersections between brands, consumption and social media in young people’s lives and the consequences for identity formation and peer acceptance.

Previous research has highlighted the importance of adolescent peer acceptance [17]. Our study found that the acceptance and rejection of brands such as Budweiser, Malibu or WKD is not entirely about the taste of the actual product, but rather it is about what the brand signifies to the consumer. For the young males involved in this study, Budweiser was believed to encapsulate maturity, masculinity and an acceptance among their peers whilst WKD was believed to signify femininity, immaturity and binge-drinking. Social group also emerged as an important factor in these discussions. Young males from ABC1 backgrounds differentiated Budweiser, associated with relaxed drinking and maturity, from WKD—which they actively distanced themselves from. Although WKD was associated with forms of femininity it was also associated with the image of the “binge” drinker which mainstream media in the UK often portrays as a lower-class activity. The groups mentioned that WKD might be purchased by parents, wishing to give their children a first taste of alcohol at a special occasion and this parental approval was deemed extremely unfashionable among the young people in the focus groups. The act of consuming alcohol in adolescence can act as a sign of rebellion or independence, coupled with a desire to emulate the lifestyle of young adults [70]. It follows that they would reject a brand such as WKD which represented parental interference and the loss of their independence [71]. The focus group participants’ desire to associate themselves with the mature, middle-class drinking culture presented by Budweiser and the social lifestyle associated with Malibu, whilst simultaneously distancing themselves from the immature, binge-drinking culture they associated with WKD, demonstrates how young people will consciously avoid certain alcohol brands which are symbolically incongruent with their desired identity [23]. These drinking cultures were also heavily gendered, with certain brands and products viewed as more socially acceptable for either males or females to be seen with and some brands being seen as inherently un-masculine. This is in line with previous research which has shown differences in consumption of different products such as beer and spirits by age and gender and has shown associations between age and gender and liking different products on social media [15,51]. The results of this study show that, even children as young as 14 and 15 are selecting products to portray a mature drinking identity that is appropriately aligned to their gender and sexuality. WKD was associated with females, gay people and the very young, all of which signify the opposite of hegemonic masculinity which is often associated with alcohol consumption [72].

A key aspect of brand identity is the persona or personality which the brand chooses to project. Brand personality is an expression of the brand’s core values and characteristics with an emphasis on human traits such as, for example, trustworthiness, excitement, stylishness or warmth [30,73]. Brands adopt a particular tone of voice, appeal to certain values and associate themselves with cultural reference points such as sport or music which are of intrinsic interest to users and encourage them to feel comfortable in the brand’s presence [30]. This was demonstrated in the example of one participant who liked to share football-related content on his Facebook page. He mentioned that he would be inclined to like the Budweiser Facebook page because of the brand’s association with football and the large amount of football-related content on Budweiser’s website and social media pages which was displayed on the examples shown to the participants. The online spaces created by alcohol brands could be seen to function as ‘glue’, bringing users together who shared similar interests or views and helping to create ‘intoxigenic digital spaces’ [69]. This creates social and emotional bonds between users which are beneficial in creating a feeling of belonging and acceptance [74]. Although these conversations may not always revolve around alcohol consumption, they reflect brand values, revealing the subtlety and complexity of branding [75]. Brands seek to create desirable associations because they contribute to brand identity and values, and also because they will be of intrinsic interest to users, who will in turn be happy to be associated with such references and share them with their social networks [61]. Previous studies have shown that much of the alcohol marketing that is targeted at younger consumers is driven by the concept of friendship, socialising and fitting in [75,76]. It became obvious that this concept was important for the young people in the groups who frequently associated Budweiser with men watching football together and Malibu with young women who like to go out socialising with their friends. Alcohol in this sense was perceived as something which could be used to bond with their peer group.

Interacting with alcohol brands on social media functions as an extension of the young peoples’ existing social reality. Social media sites allow the user to create and share content which is meaningful to them and brands become part of this. As the results of this study demonstrate, those brands which were viewed as socially acceptable were more attractive to the young people and this influenced their likelihood of interacting with the brand’s social media profile. Interactions like this are central to how much marketing circulates on social media. Digital alcohol marketing aims to promote interaction between brands and social media users, as well as user-to-user conversations among potential consumers [39]. Alcohol marketers will post an item of content on their official page but what is more important to the brand is how far that content reaches out into users’ networks and how many times that item of content is displayed [47]. By appealing to certain values and creating desirable brand associations, alcohol brands are able to craft a personality and convey to users the impression that their online content was not from faceless corporations but from friends with similar interests, making it easier for users to pass on the brand messages within their social network and thereby to extend the reach of the brand’s marketing and to lend it an authenticity and persuasiveness [43]. This blurs the distinction between user and commercially generated content, enabling brands such as Budweiser to perform in ways online that existing ‘offline’ regulations explicitly prohibit. For example, the young people in this study closely associated Budweiser not only with masculinity and sport but also with a socially desirable form of underage drinking. The act of ‘liking’ the brand helps the brand to travel among the social networks of these young people, lending credibility and authenticity to the marketing messages in a way that would not be tolerated in an ‘offline’ environment [28]. This raises important points regarding the regulation of such content on social media. Strategies are required to reduce the exposure to, and potential impact of, alcohol marketing on social media sites, and ensure these sites are neither accessible to nor targeting underage social media users [76]. The participants in this study felt that any age restrictions employed by social media sites were ineffective at restricting the amount of alcohol marketing they saw on social media. These restrictions vary between social media sites; Facebook, for example, restricts access to official alcohol marketing pages to those over the age of 18 in the UK. However, our findings that young people aged 14–17 are able to access alcohol marketing are consistent with previous studies [40,77]. Previous research has also found that alcohol marketing pages on social media sites are particularly appealing to those under the legal drinking age [78].

Social media sites are a vital part of youth culture and maintaining a presence on social media facilitates participation in a peer network and helps young people develop their social identity [49]. The importance of projecting the right sort of image online was reflected in our study in participants’ likelihood of engaging with alcohol brands online. Social media provides a platform for young people to establish and explore self-identity through interaction with others by providing other users with feedback on their own behaviour and allowing them to monitor the behaviour of their peers and how others react to this behaviour [79]. The young people were conscious that they would be openly endorsing brands to their peers and subjecting themselves to almost instantaneous feedback through likes and comments which can either publicly validate or criticise their choices [63].

### Limitations and Scope for Future Work

This research was conducted in one area of the UK, Central Scotland, and in this respect findings may not be generalizable to young people from other parts of the UK or other countries. Previous work investigating the alcohol consumption of young people in Scotland has shown that some differences exist between Scotland and the rest of the UK [80]. However, previous studies with more diverse groups of young people support our findings with regard to the value attached to mainstream food brands [81] and the importance of brands to peer acceptance [82]. In addition, a similar study conducted in England [51] found that alcohol marketing on social media was entangled with young people’s cultural and identity-making practices in ways that created greater relevance and meaning. Friendship groups were used in order to relax the participants and encourage them to speak more freely. Friends are more likely to disagree with each other and to know when someone is not telling the truth [83]. However, it is acknowledged that by including groups of different friends in the same group the young people may have attempted to inflate their drinking practices in order to appear more mature than the other groups. In addition, some individuals may have influence over their friends and this may have shaped the discussion.

All participants were asked about their frequency of alcohol consumption during recruitment. All those recruited had at least consumed alcohol at least once within the last year. This means that the study did not take into account the views of young people who have not consumed alcohol and further research could explore whether these young people would attach similar meanings to alcohol brands or whether another form of consumption was used to replace alcohol brands [18,19]. However, inclusion of non-drinkers in this study could have contradicted the views of drinkers, particularly in relation to how interacting with alcohol brands on social media impacts on young people. Participants were not asked to clarify their level of alcohol consumption during the focus groups and it is acknowledged that this would have provided a useful context for the discussions and allowed for a richer analysis of the data. Much of the discussion focused on the use of Facebook. However, many young people are reported to be moving away from Facebook and using other social media sites such as Whatsapp and Snapchat. Future research should explore what role these sites play in identity formation and how alcohol use is portrayed on these sites.

## 5. Conclusions

Young people’s dependence on social media as a means of everyday social interaction has vast implications for marketers. The marketing of alcohol brands plays an increasingly integral part of young people’s everyday lives at a stage where they are striving to achieve their own personal identity [16]. The advent of social networking means that corporations can have direct access to, and form part of, a young person’s social reality. Brands are able to constantly reinforce their values, cementing the brand identity in consumers’ minds and encourage intimate forms of connection and relationship with, and between, consumers online. Current marketing regulations aimed at protecting vulnerable groups such as young people can be seen as ineffective because they seek to apply existing codes to the online environment rather than addressing the unique challenges posted by social media [28,39,77]. Understanding how young people perceive alcohol brands, the importance of social networking sites in communicating that identity to their peers and the role that alcohol brands play in adolescent identity formation is an important first step to reforming alcohol marketing regulations. 

These insights deepen our understanding of how young people establish their identities through alcohol consumption and how alcohol marketing is connected to young peoples’ concerns about image and peer group dynamics.

## Figures and Tables

**Table 1:** Details of focus group participants.

Group Number	Location	Gender	Age	Socio-Economic Group ^1^
Group 1	Edinburgh	Male	14–15	ABC1
Group 2	Edinburgh	Male	16–17	C2DE
Group 3	Edinburgh	Female	14–15	ABC1
Group 4	Edinburgh	Female	16–17	C2DE
Group 5	Glasgow	Male	14–15	C2DE
Group 6	Glasgow	Male	16–17	ABC1
Group 7	Glasgow	Female	14–15	C2DE
Group 8	Glasgow	Female	16–17	ABC1

^1^ Standard classifications used: AB (Higher & intermediate managerial, administrative, professional occupations); C1 (Supervisory, clerical & junior managerial, administrative, professional occupations); C2 (Skilled manual occupations); DE (Semi-skilled & unskilled manual occupations, unemployed and lowest grade occupations) [64].

**Table 2:** Details of participants’ social media use.

	Visited Several Times a Day	Visited Once a Day	Visited Several Times a Week	Visited Once a Week	Visited Once a Month	Visited Less than Once a Month
Facebook	36	10	1	0	0	1
Twitter	12	5	8	1	0	0
YouTube	19	15	12	1	0	0
Tumblr	6	4	21	3	0	0
Pinterest	0	0	0	0	6	2
Other	15	12	6	0	9	2

**Table 3:** Details of alcohol brands (as of 10 March 2014).

Brand Name	Type of Beverage	% ABV	Facebook Fans (Likes)	Twitter Followers	YouTube Subscribers
Budweiser	Beer	4.8	11,590,403	4358	1136
Malibu	Rum	21	2,076,725	9431	1456
WKD	Pre-mixed drink (alcopop)	4	284,944	11,200	n/a

ABV: Alcohol by Volume.

## Appendix A

**FOCUS GROUP TOPIC GUIDE**

Participants: 8 single sex friendship groups comprising of 14–15-year olds and 16–17-year olds (6 participants per group). The discussion will cover the topic areas outlined below, but will vary in focus in accordance with participant’s range of experience. These topics should not been seen as a rigid structure, but as a checklist, providing participants with the freedom to express their views and experiences.

**1. Begin with general discussion on social network sites (explore spontaneous thoughts and experiences of digital media**

- To begin with, what do you understand by the term ‘social media’?
- Do any of you use social media? How often?
- What is the main reason(s) for using social media?
- Which sites do you use and why do you think they are popular?
- Do you ever go on commercial pages? What sort of things do you look at?
- Do you ever ‘like’ commercial pages or follow commercial tweets? What pages/tweets? Why?
- Do you ever go on alcohol brand pages? What brands? Why?
- Do you ever ‘like’ alcohol brand pages?
- Do you see a lot of alcohol marketing online? What have you seen? What do you think about this?
- Do you ever go on ‘fan’ pages (unofficial alcohol pages)? What pages? Why?
- Do you feel there is a difference between official brand pages and ‘fan’ pages?

**2. Descriptions of alcohol brands on social network sites (explore participants’ reaction to alcohol marketing on social network sites and how alcohol brands feature in online conversations)**

- Do you or your friends ever talk about alcohol brands online? If so, how and where?
- Do you ever talk about specific brands? What brands?
- Have you ever seen anyone posting pictures of alcohol brands or alcohol use on their profile? Any examples? How often?

**Show examples of branded SNS pages using I Pad**

- Reactions to specific examples of online alcohol marketing on SNS
- Have any of you seen these pages before?
- What sort of person would go on this page?
- Do participants find any of the examples appealing? If so, why?
- Could you rank the SNS pages in order from most appealing to least appealing? Why are some appealing and others not? What would make you likely to go on this page (competitions, giveaways etc.)?

**3. General descriptions of alcohol packaging and labelling (explore reactions, attitudes and descriptions)**

- Can you think of anything that you particularly liked or that you particularly remember? What was it that you liked about it?
- Are there any types of products for which the packaging stands out? What? Why? What features?
- Are there any types of products where a great deal of effort has been put into the packaging? What? Why? What features?

Think about alcohol products and the way they are packaged. The shape, the colours, the label etc.

- Do you think packaging is important for alcohol brands? Why?
- Can you describe any examples of alcohol packaging for me? Any specific brands which come to mind?

I’ve got some examples of alcohol packaging to show you. **Place items on table**.

- Have you seen any of these items before? Where? What situation?
- What springs to mind when you see this pack? What words would you use to describe this pack?
- Imagine this pack is a person—can you describe them? What do they look like/wear/like to do?

**Ordering (use showcards)**

1. Order from most appealing to least appealing:
  Discuss order/reasoning behind order. Target products—why X is different from Y
  What is it you like/dislike about them?
  What are the good/bad features?
2. Order for someone like me/not like me
3. Order from pleasant taste/unpleasant taste
4. Order from most harmful (strongest)/least harmful (weakest).

- Choose your favourite pack. Why have you chosen this?
- Choose your least favourite pack. Why have you chosen this?
- What is it about the pack that makes you feel that way?

**4. Reaction to interactivity of the packaging and labelling** (participants’ reaction to QR codes, web addresses etc. and their likelihood of further engagement with brands online)

- Have any of you noticed any web addresses, Facebook or Twitter addresses on the packaging?
- Would you ever be likely to go to these addresses? Why/why not?
- Do any of you own smartphones?
- Do any of you know what QR codes are?
- Have you seen these before? Where?
- Have any of you used QR codes before?
- Have you ever seen them on alcohol packaging?

**Demonstrate examples of QR codes (Dragon Soup, Malibu and Bulmers).**

- What do you think of this type of technology?
- Do you find any of the examples appealing? What? Why?
- Would you be likely to scan these codes in the future? Why? (curiosity, competitions etc.) Who do you think would?
- Do any if you know what augmented reality is? Have any of you used Blippar, Augment or other A.R. apps before?
- Have you seen these before? Where?
- Have you ever scanned anything with A.R packaging?
- Have you ever seen them on alcohol packaging?

**Demonstrate examples of AR packaging. (WKD Blippar, Youtube vids on I Pad)**

- What do you think of this type of technology?
- Do you find any of the examples appealing? What? Why?
- Would you be likely to scan this packaging in the future?
- Who do you think would be likely to use this kind of technology? Why?
